# Detection of *Leishmania infantum* DNA mainly in *Rhipicephalus sanguineus* male ticks removed from dogs living in endemic areas of canine leishmaniosis

**DOI:** 10.1186/1756-3305-5-98

**Published:** 2012-05-21

**Authors:** Laia Solano-Gallego, Luca Rossi, Anna Maria Scroccaro, Fabrizio Montarsi, Marco Caldin, Tommaso Furlanello, Michele Trotta

**Affiliations:** 1 Department Pathology and Infectious Diseases, Royal Veterinary College of London, United Kingdom; Departament de Medicina i Cirugia Animals, Universitat Autonoma de Barcelona, Cerdanyola, Spain; 2 Clinica Veterinaria Corridori-Rossi-Marioni, Grosseto, Italy; 3 Laboratorio e Clinica Veterinari San Marco, Padua, Italy; 4 Dipartimento di Ecopatologia, Istituto Zooprofilattico Sperimentale delle Venezie, Padua, Italy; 5 Departament de Medicina i Cirugia Animals, Facultat de Veterinària, Universitat Autònoma de Barcelona, Cerdanyola, Barcelona, 08193, Spain

**Keywords:** *Leishmania infantum*, *Rhipicephalus sanguineus* ticks, PCR, Dog

## Abstract

**Background:**

Sand flies are the only biologically adapted vectors of *Leishmania* parasites, however, a possible role in the transmission of *Leishmania* has been proposed for other hematophagous ectoparasites such as ticks. In order to evaluate natural infection by *Leishmania infantum* in *Rhipicephalus sanguineus* ticks, taking into account its close association with dogs, 128 adult *R. sanguineus* ticks removed from 41 dogs living in endemic areas of canine leishmaniosis were studied.

**Methods:**

Individual DNA extraction was performed from each tick and whole blood taken from dogs. Dog sera were tested for IgG antibodies to *L. infantum* antigen by ELISA and *L. infantum* real-time PCR was performed from canine whole blood samples and ticks.

**Results:**

*Leishmania infantum* PCR was positive in 13 ticks (10.1%) including one female, (2.0%) and 12 males (15.2%), and in only five dogs (12.2%). Male ticks had a significantly higher infection rate when compared to female *R. sanguineus*. The percentage of *L. infantum* seroreactive dogs was 19.5%. All but two PCR positive dogs were seroreactive. *Leishmania infantum* PCR positive ticks were removed from seropositive and seronegative dogs with a variety of PCR results.

**Conclusions:**

This study demonstrates high prevalence of *L. infantum* DNA in *R. sanguineus* ticks removed from *L. infantum* seropositive and seronegative dogs. The presence of *L. infantum* DNA was detected mainly in male ticks possibly due to their ability to move between canine hosts and feed on several canine hosts during the adult life stage. Additional studies are needed to further explore the role of *R. sanguineus* ticks and in particular, male adults, in both the epidemiology and immunology of *L. infantum* infection in dogs in endemic areas.

## Background

Canine leishmaniosis due to *Leishmania infantum* is a major zoonotic disease endemic in more than 70 countries in the world. It is present in regions of southern Europe, Africa, Asia, South and Central America [1]. It has been estimated, based on seroprevalence studies from Italy, Spain, France and Portugal that 2.5 million dogs in these countries are infected [2]. Furthermore, *L. infantum* infection is spreading north in Europe [3] and has reached the foothills of the Alps in Northern Italy [4]. Dogs are the main reservoir for this infection and the disease can be fatal sometimes if not treated in people and dogs.

*Leishmania infantum* is a diphasic parasite that completes its life cycle in two hosts, a sand fly, which harbors the flagellated extracellular promastigotes and a mammal where the intracellular amastigote parasite forms develop. Female sand flies of some species of the genus *Phlebotomus* (Old World) are the proven vectors of *L. infantum* transmission in humans and dogs [5]. However, other less common transmission routes have been proven in dogs. The transmission of *L. infantum* through blood products has been reported in dogs that received blood transfusions from infected donors in North America [6,7]. Transmission of infection by infected canine blood products has been documented and is of special concern in areas where blood donors may be carriers of infection [8,9]. Vertical in-utero transmission from dam to its offspring has been documented [10,11] and venereal transmission has been also reported in dogs [12]. Direct dog-to dog transmission without involvement of a hematophageous vector has been suspected in some cases of infection in areas where vectors of the disease are apparently absent [13]. In addition, ticks and fleas have been proposed as alternative vectors of *L. infantum* transmission [14,15].

*Rhipicephalus sanguineus*, known as the brown dog tick, is an ectoparasite of domestic dogs found almost worldwide, mainly in tropical and temperate climates [16]. *Rhipicephalus sanguineus* is a three-host tick that feeds primarily on dogs and occasionally on a diverse range of wild and domestic animals, including humans [17,18].

*Rhipicephalus sanguineus* ticks are known vectors and reservoirs of canine pathogens such as *Babesia vogeli* and *Rickettsia conorii*. Little is known of the relationship between *R. sanguineus* ticks and certain pathogens that they might transmit. The complete life cycle of *Hepatozoon canis* in *R. sanguineus* tick has recently been described [19]. Studies of this nature are lacking for other pathogens. For instance, the mechanisms involved in the interaction between *R. sanguineus* and *L. infantum* are largely unknown. Although, it is suspected that *R. sanguineus* ticks might be involved in the transmission of *L. infantum*[15], the role of the brown dog tick in the epidemiology of *L. infantum* infection remains uncertain and is an issue of great importance for dogs and humans [20].

In order to evaluate natural infection by *L. infantum* in *R. sanguineus* ticks, taking into account its close association with dogs, *R. sanguineus* ticks removed from dogs living in endemic areas of canine leishmaniosis were studied. In this context, the aims of the present study were to detect *L. infantum* DNA in ticks collected from dogs living in endemic areas of leishmaniosis and to evaluate the relationship between *Leishmania* infection in ticks and dogs and the pattern of *Leishmania* DNA detection in ticks.

## Methods

### Dogs and ticks

Fifty-eight ticks removed from 26 apparently healthy dogs and two sick dogs living in an outdoor shelter in an endemic area of canine leishmaniosis (Grosseto, Central Italy) were studied between March to May 2006 (Group 1). In addition, a total of 70 ticks were removed from 13 dogs with clinical signs and laboratory findings compatible with tick-borne diseases from April to November 2007 from veterinary clinics throughout Italy [21] (Group 2). After collection, the ticks were placed in a tube with 75% ethanol, and sent to laboratory San Marco where they were immediately stored at −20°C. Later, the ticks were dried at room temperature and morphologically identified [22]. The mean number of ticks collected per dog and standard deviations were 3.1 ±3.3 and the range of ticks per dog was from 1 to 15. K_3_EDTA blood and sera were collected from all dogs studied from both groups and stored until use at −20°C. Different breeds and mixed-breed of dogs were studied in both groups.

The mean age ± SD of dogs in Group 1 was 5.05 ± 2.13 years. The information on age was available only for 17 dogs. Sixteen were females and 12 were males. All but one female dog were neutered. The mean age ± SD of dogs in Group 2 was 5.7 ± 4.7 years. The information about age was available only for 9 dogs. Six were females and three were males. Gender was not available for all dogs.

### DNA extraction

The ticks stored in 75% ethanol were dried, washed with PBS and left overnight in PBS at 4°C to eliminate ethanol. The DNA was isolated from individual ticks by using the High Pure PCR template preparation kit (Roche, Mannheim, Germany) according to the manufacturer’s instructions with some modifications. The ticks were mechanically crushed with a sterile micropestle, suspended in 200 μL of tissue lysis buffer and 40 μL of proteinase K (100 μg/mL) and incubated overnight at 65°C. The final elution volume was 100 μL for each sample.

DNA extraction from canine whole blood was performed by using the High Pure PCR template preparation kit (Roche, Mannheim, Germany). 200 μL of whole blood was mixed with 200 μL of binding buffer with proteinase K (100 μg/mL) and incubated for 1 h at 72°C. The DNA extraction was then carried out according to the manufacturer’s instructions. The final elution volume was 50 μL for each sample.

Cultured *L. infantum* (MHOM/FR/78/LEM75) zymodeme MON-1, *Leishmania tropica* (MCAN/MA/90/LEM2007) zymodeme MON-102 and *Leishmania braziliensis* (MCAN/BR/81/RICO) zymodeme MON-43 reference strains were studied. *In vitro*-cultivated *L. infantum* promastigotes in the logarithmic phase of growth were washed three times by centrifugation for 5 min at 1500 g in sterile PBS and counted in a hemocytometer by using a light microscope. Genomic DNA was extracted from 200 μL of 73X10^6^ promastigotes/mL as previously described [23].

### Conventional PCR for tick mitochondrial 16 S rRNA gene

The efficiency of tick DNA extraction was evaluated by amplification of the tick mitochondrial 16 S rRNA gene (ribosomal DNA [rDNA]) using tick-specific primers in a conventional PCR: TQ16S-1 F 5′CTGCTCAATGATTTTTTAAATTGCTGTGG 3′ and TQ16S-2R 5′ ACGCTGTTATCCCTAGAG 3′, as previously described [24].

Each reaction was carried out in 50 μL volume containing 0.5 μmol/μL of each oligonucleotide primer, 2.5 mM of each dNTP (5PRIME GmbH, Hamburg, Germany), 5 μL of 10× PCR buffer, 1 U of *Taq* DNA polymerase (5PRIME GmbH, Hamburg, Germany) and 5 μL of the DNA.

PCR was performed in a thermocycler (Applied Biosystem, Europe) with 1 cycle of denaturation (8 min, 94°C), followed by 10 cycles of denaturation (1 min, 92°C) annealing (1 min, 48°C) and extension (1 min 30 s, 72°C) then 32 cycles of denaturation (1 min 92°C), annealing (1 min, 54°C), extension (1 min 30 s, 72°C) and a final extension step (10 min, 72°C) as previously described [24]. DNA electrophoresis was carried out in 2.2% FlashGel System (Lonza, Rockland, ME USA), and DNA fragments were visualized.

### *Leishmania infantum* real-time PCR and sequencing

Real-time PCR for *L. infantum* DNA detection was performed in all the tick and canine samples.

We used primer and probe sequences specific for the *L. infantum* kinetoplast mini-circle (TIBMOLBiol, Genova, Italy) as described [23]. Real-time PCR was performed by using LightCycler FastStart DNA Master^PLUS^ Hybridization Probes (Roche, Mannheim, Germany) using LightCycler version 3.5.17 instrument (Roche, Mannheim, Germany). Positive and negative controls were used in all runs [23]. *Leishmania tropica* and *L. braziliensis* DNA were not detected by qPCR hybridization probes while *L. infantum* DNA was detected.

One positive tick sample was sequenced. The kDNA real-time PCR product was sequenced at TIB MOL Biol (Genova, Italy). Sequences were compared with sequences deposited in GenBank using BLAST hosted by the National Center for Biotechnology Information, National Institutes of Health, USA (http://www.ncbi.nlm.nih.gov).

### ELISA

Canine serum anti-leishmanial antibodies were determined by ELISA, using sonicated crude *L. infantum* antigen as previously described [25]. Briefly, dog sera were diluted to 1:400 and incubated in *L. infantum* antigen-coated plates (20 μg/mL) for 1 hour at 37°C. The plates were then washed with 0.05% Tween 20 in phosphate-buffered saline (PBS) and incubated with Protein A conjugated to horseradish peroxidase (1:10,000 dilution; Sigma) for 1 hour at 37°C. Plates were washed again with 0.05% PBS-Tween 20. The plates were developed by adding the substrate solution *ortho*-phenylene-diamine (SIGMA FAST OPD, Sigma). The reaction was stopped with 50 μl of 3MH_2_SO_4_. Absorbance values were read at 492 nm in an automatic microELISA reader (PROGRAMMABLE MPT READER DV 990BV4, N.T. Laboratory, Italy). All determinations included the serum from a sick dog with a confirmed infection as positive control (calibrator) and serum from a healthy dog as a negative control. The result was quantified as units (U) related to a positive canine serum used as a calibrator and arbitrarily set at 100 U. The cutoff was established at 14 U (mean + 4 SD of values from 32 dogs from non-endemic areas) and the results above this value were considered positive.

### Statistical analysis

Differences among groups were analyzed by means of Chi-square with Yates correction if needed and Fisher’s exact test. A *p <* 0.05 was considered significant.

## Results

All ticks from both groups were morphologically identified as adult *R. sanguineus* ticks. A 320 bp fragment of the tick mitochondrial 16 S rRNA gene was amplified in all DNA tick samples from both groups. Serological and molecular results from the dogs are shown in Table 1. The molecular results from ticks are shown in Table 2. The closest matches to the amplified k-DNA product were always *L. infantum* with an identity of 95% over the 56 bp amplified sequence.

**Table 1:** ***Leishmania infantum*seroprevalence and PCR results in dogs from groups 1 and 2**

	**Serology**	***L. infantum*PCR**
Group 1	6/28 (21.4%)	4/28 (14.2%)
Group 2	2/13 (15.3%)	1/13 (7.7%)
Total	8/41 (19.5%)	5/41 (12.2%)

**Table 2:** **Results of*L. infantum*PCR in male and female ticks removed from dogs belonging to group 1 and 2**

	***L. infantum*PCR in ticks**		
	**Females**	**Males**	**Total**
Group 1	1/17 (5.8%)	10/41 (24.3%)	11/58 (18.9%)
Group 2	0/32 (0%)	2/38 (5.2%)	2/70 (2.8%)
Total	1/49 (2.0%)	12/79 (15.2%)*	13/128 (10.1%)

*Fisher’s exact test; P = 0.0166.

### Group 1

*Leishmania infantum* PCR was positive in eleven ticks (18.9%), including one female (5.8%) and 10 males (24.4%), and in only four dogs (14.2%). No statistical difference was found between the percentages of positive female and male ticks (Fisher’s exact test; P = 0.1482). The percentage of *L. infantum* seroreactive dogs was 21% (6/28). All but one PCR positive dog was seroreactive. *Leishmania infantum* PCR positive ticks were removed from both seropositive and PCR-positive dogs (n = 2), from both seronegative and PCR-negative dogs (n = 3), from one seropositive and PCR negative dog and from one seronegative and PCR positive dog. The only PCR positive female tick was removed from a sick seropositive and PCR positive dog.

### Group 2

*Leishmania infantum* PCR was positive in only two male ticks (5.2%) and in only one dog (7.7%). No statistical difference was found between the percentages of positive female and male ticks (Fisher’s exact test; P = 0.4965). The percentage of *L. infantum* seroreactive dogs was 15.3%. *Leishmania infantum* PCR positive ticks were removed from two seronegative and PCR-negative dogs.

### Both groups

When results from the two dog groups were combined, *L. infantum* PCR was positive in 13 ticks (10.1%) including one female (2.0%) and 12 males (15.2%) and in only five dogs (12.2%). Statistical difference was found between the percentages of total *Leishmania* PCR positive female and male ticks when all ticks studied from both groups were taken into account (Fisher’s exact test; P = 0.0166; chi-square with Yates correction = 4.380; P = 0.0364). The percentage of *L. infantum* seroreactive dogs was 19.5%. All but two PCR positive dogs were seroreactive. *Leishmania infantum* PCR positive ticks were removed from both seropositive and PCR-positive dogs (n = 2), from both seronegative and PCR-negative dogs (n = 5), from one seropositive and PCR negative dog and from one seronegative and PCR positive dog. *L. infantum* PCR-positive and negative ticks were removed from the same dog (n = 7). 

## Discussion

Ticks and fleas have been proposed as alternative vectors of *L. infantum* transmission whether biologically [26] or mechanically [14,15]. Therefore, it is important to evaluate the prevalence of *L. infantum* infection in ticks to determine whether they could represent a source of infection for dogs living in endemic areas [20]. In the present study, *L. infantum* DNA was detected in *R. sanguineus* ticks. The percentage of ticks positive for *L. infantum* DNA using PCR was lower but similar to that found in a study performed in Brazil [27]. Conversely, the *L. infantum* DNA detection rate was much higher (50%) in ticks from Brazil [28]. It is important to highlight that previous studies evaluated ticks removed from seropositive infected dogs that are likely to have a higher parasite load and, therefore, overestimating the prevalence of *Leishmania* DNA detection in ticks [27,28] due to the fact that canine *Leishmania* infection increases the probability of finding a positive tick [20]. Instead, seronegative and seropositive dogs from a dog population living in an endemic area of leishmaniosis with seroprevalence similar to that described in previous literature from southern European countries [29,30] were studied in this manuscript. Interestingly, a study performed in Sardinia failed to detect *Leishmania* DNA by conventional PCR in *R. sanguineus* ticks removed from dogs [31]. However, clinical and parasitological information about the dog population studied was lacking [31]. Curiously, the percentages of *Leishmania* infection in ticks appear to be much higher than the percentages observed in sandflies with natural infection, which are usually lower than 1% [32]. Future studies should be carried out in endemic areas to assess the prevalence of *Leishmania* infection in large numbers of *R. sanguineus* ticks collected from dogs.

Interestingly, this study demonstrates the presence of *L. infantum* DNA mainly in male *R. sanguineus* ticks when removed from a dog population in endemic areas. In addition, the majority of positive male ticks were removed from seronegative and PCR negative dogs. It has been shown that *R. sanguineus* male ticks readily migrate between infested, co-housed dogs in close contact due to the fact that they intermittently feed prior to and between mating on a host [33]. Therefore, they may serve as a source of *Leishmania* infection when they move directly between dogs or survive in the environment long enough to eventually find another canine host [33]. In addition, movement of adult ticks between dogs represents a naturally occurring form of interrupted feeding, a strategy which has been shown to shorten the feeding time necessary to allow transmission of pathogens [33]. These findings might suggest the possibility of cross-transfusion of infected blood between dogs by male *R. sanguineus* ticks when feeding. However, evidence of transmission of *Leishmania* infection by tick bites was reported experimentally only in two dogs [20,26]. Further studies need to elucidate the potential of *Leishmania* transmission to dogs via male tick bites.

There is little information about the development of *Leishmania* parasites in the tick. So far, transtadial and transovarial transmission of *L. infantum* DNA in *R*. *sanguineus* ticks have been demonstrated [34] and *Leishmania* DNA has been detected in salivary glands from ticks [34,35]. It remains unknown if promastigote forms can develop in the tick or if only amastigote forms migrate and invade several tissues of ticks including tick gut, ovaries or salivary gland. We propose that *Leishmania* amastigote forms could be transmitted to dogs by male *R. sanguineus* ticks that have fed on infected canine blood. However, further studies need to be performed to understand the contribution of different stages of *R. sanguineus* in the transmission of *Leishmania* to dogs [34,35]. Indeed, there is no definitive evidence that, at least, ticks in their different life stages can harbour viable *L. infantum*, because no live parasites have been cultivated from ticks *in vitro* or *in vivo* models. Another main problem to support the role of ticks in *L. infantum* transmission is the evidence that, till now, there are no scientific demonstrations of *Leishmania* biological forms (amastigote or promastigote forms) in the ticks, as assessed by dissection and morphological studies [20,28].

## Conclusions

This study demonstrated a high prevalence of *L. infantum* DNA in *R. sanguineus* ticks removed from dogs seropositive or seronegative for *L. infantum*, and in dogs positive or negative for the presence of *L. infantum* DNA. *Leishmania infantum* DNA was found mainly in male ticks. Additional studies are needed to further explore the role of *R. sanguineus* ticks and in particular, male adults, in both the epidemiology and immunology of *L. infantum* infection in dogs in endemic areas.

## Competing interests

The authors declare that they have no competing interests.

## Authors’ contributions

LSG designed the study, contributed with data analysis and interpretation and wrote the manuscript. LR collected ticks and canine blood samples. AS performed DNA extraction. FM morphologically identified the ticks. MC and TF contributed with the revision of the manuscript. MT performed DNA extraction, ran the conventional and real-time PCRs, contributed with data analysis and interpretation and revision of the manuscript. All authors read and approved the final manuscript.
